# *MaCsbD* Mediates Thermotolerance and UV-B Resistance in *Metarhizium acridum* by Regulating DNA Repair, Antioxidant Defense, and Protective Metabolites

**DOI:** 10.3390/jof11120838

**Published:** 2025-11-27

**Authors:** Xinyu Li, Ke Li, Yuxian Xia

**Affiliations:** 1 Genetic Engineering Research Center, School of Life Sciences, Chongqing University, Chongqing 401331, China; 202326021002@stu.cqu.edu.cn (X.L.); 15893649836@163.com (K.L.); 2 Chongqing Engineering Research Center for Fungal Insecticide, Chongqing 401331, China; 3 Key Laboratory of Gene Function and Regulation Technologies Under Chongqing Municipal Education Commission, Chongqing 401331, China

**Keywords:** fungal stress response, CsbD-like protein, stress tolerances, trehalose, melanin

## Abstract

Although CsbD-like proteins have been studied extensively in bacteria, their functions in eukaryotes remain largely uncharacterized. Our study investigated the CsbD homolog MaCsbD in the entomopathogenic fungus *Metarhizium acridum* and uncovered its importance for coping with environmental stress. Loss of *MaCsbD* resulted in delayed conidial germination, reduced conidial yield, and heightened sensitivity to UV-B irradiation and heat shock. The mechanism analysis revealed that the absence of *MaCsbD* led to a decline in DNA repair capacity, a weakening of the antioxidant defense mechanism, and a reduction in the induction of heat shock proteins. The determination of the accumulation levels of protective metabolites, melanin, and trehalose in the conidia showed that their contents were significantly decreased. Phylogenetic analysis further revealed that CsbD-like domains are conserved across fungi, suggesting an evolutionary role in stress adaptation. Virulence against locusts was unchanged, indicating that *MaCsbD* primarily supports abiotic stress tolerance rather than pathogenicity. *MaCsbD* is therefore required for robust fungal stress responses and identifies a potential target for improving the field performance of fungal biocontrol strains.

## 1. Introduction

Entomopathogenic fungi are a key factor in sustainable agriculture, providing an eco-friendly alternative to chemical pesticides by targeting specific pests while protecting biodiversity [1,2,3]. Among these, *Metarhizium acridum* (*M. acridum*) is particularly valued for locust control due to its strict host specificity and minimal ecological impact [4,5]. Despite its ecological advantages, practical applications face limitations stemming from inconsistent field efficacy, delayed mortality rates, and susceptibility to abiotic stressors such as ultraviolet (UV-B) radiation and heat shock [6]. Improving conidial production and stress resilience is therefore critical to enhancing its biocontrol performance [7].

Stress tolerance in filamentous fungi relies on complex protective mechanisms, ranging from metabolic reprogramming to specialized stress-related proteins [8,9]. Thermotolerance in *Aspergillus* and *Penicillium* species, for instance, is mediated by rapid induction of heat shock proteins (Hsp70/Hsp90) that maintain proteostasis under thermal stress [10,11]. Similarly, UV-B resistance involves enhanced nucleotide excision repair (NER) through upregulation of rad family genes [12]. The anti-UV-B effects of *Rad2*, *Rad14*, and *Rad26* in *Metarhizium rosenii* are primarily mediated by the nucleotide excision repair pathway [13]. Understanding these adaptive mechanisms provides critical insights for improving fungal biocontrol agents’ field performance.

CsbD-like proteins were initially identified in *Bacillus subtilis* as stress response factors controlled by σ^B^ and have since been implicated in tolerance to harsh conditions such as bile salts and freeze-drying in bacteria [14,15]. In Group B *Streptococcus* (GBS), the *CsbD* gene functions as a bile salt tolerance response factor. It induces the transcription of ABC transporter genes to expel bile salts, enhancing the survival ability of bacteria in a bile salt environment [16]. Although the functions of the CsbD-like proteins in bacteria have gradually become clear, their biological roles in eukaryotes have not yet been studied. Therefore, studying CsbD-like proteins in eukaryotes is crucial for understanding their biological functions and regulatory mechanisms. Based on the conserved role of CsbD in general stress tolerance across bacteria, we reasoned that *MaCsbD* would function in *M. acridum* by combating core abiotic stress damages—namely, DNA lesions, ROS, and homeostatic imbalance. This logic led us to test its role in regulating DNA repair, ROS scavenging, and the production of protective metabolites like trehalose, which are critical for this fungus’s survival in harsh environments.

In this study, *MaCsbD* (J3458_004571) was identified as a CsbD-like protein in *M. acridum*. The objectives of this study include: (1) determining the effect of *MaCsbD* on the melanin and trehalose synthesis of *M. acridum*; (2) evaluating the impact of *MaCsbD* deletion on the germination, yield, and stress resistance of conidia under heat shock and UV-B radiation conditions; and (3) analyzing the differences in the mechanism by which *MaCsbD* regulates heat tolerance and UV-B adaptation in *M. acridum*. Unlike CsbD in bacteria, which enhances bile salt tolerance, the *MaCsbD* gene enhances the tolerance of *M. acridum* to UV-B radiation and heat shock. Additionally, knocking out the *MaCsbD* gene in *M. acridum* results in delayed germination and reduced yield of conidia, but does not affect its pathogenicity. It is noteworthy that the sensitivity of conidia to heat shock and UV-B increases. Our research results indicate that *MaCsbD* controls melanin synthesis through different mechanisms and regulates heat tolerance and UV-B adaptation.

## 2. Materials and Methods

### 2.1. Strains and Growth Conditions

The wild-type (WT) strain of *M. acridum*, CQMa102 (deposited at the China General Microbiological Culture Collection Center, CGMCC No. 0877), was used. *M. acridum* was routinely cultured on 1/4 strength Sabouraud’s dextrose agar supplemented with 1% yeast extract (1/4 SDAY; 1% glucose, 0.25% peptone, 0.5% yeast extract, 1.8% agar) [17]. The medium pH was adjusted to 6.0–6.5 before autoclaving. Plasmid propagation and recombinant clone screening were performed in *Escherichia coli* DH5α (TransGen Biotech, Beijing, China) [18]. Fungal transformation was carried out using *Agrobacterium tumefaciens* AGL-1 (Weidi Biotech, Shanghai, China) through the *Agrobacterium*-mediated transformation (ATMT) method.

### 2.2. Bioinformatic Analyses

All homologous genes and protein sequences of *MaCsbD* (GenBank entry: J3458_004571 (gene) and XP_007807437.1 (protein)) were obtained from NCBI (https://www.ncbi.nlm.nih.gov/, accessed on 15 January 2024). Expasy through the online website (https://web.expasy.org/protparam/, accessed on 15 January 2024) was used to predict protein molecular weight and isoelectric point, using the NCBI analysis domain (https://www.ncbi.nlm.nih.gov/, accessed on 15 January 2024) for protein structure prediction. DNAMAN v7.1 software was used for multiple sequence alignment, and the phylogenetic tree was constructed using the neighbor-joining method in the MEGA v 7.0 software [19].

### 2.3. Gene Deletion and Complementation

We used pK2-PB with the phosphinothricin resistance gene and pK2-sur with the chlorsulfuron ethyl resistance gene to construct knockout and complementary strains, respectively [20]. Primers *MaCsbD*-LF/*MaCsbD*-LR and *MaCsbD*-RF/*MaCsbD*-RR were designed to amplify the upstream and downstream flanking sequences of the *MaCsbD*, respectively, by PCR. The amplified upstream and downstream fragments were sequentially ligated into the pK2-PB vector to generate the *MaCsbD* knockout construct, utilizing the vector’s homologous recombination arms. The construct was subsequently introduced into the wild-type CQMa102 strain using the *Agrobacterium*-mediated method. Primary transformants were selected on Czapek-Dox agar (Sigma, St. Louis, MO, USA) supplemented with 500 μg/mL phosphinothricin. Putative *MaCsbD* knockout mutants (Δ*MaCsbD*) were confirmed by junction PCR with locus-specific primers and subsequent Sanger sequencing. For genetic complementation, the native promoter and full-length open reading frame (ORF) of *MaCsbD* were cloned into the pK2-Sur vector under endogenous regulatory elements. The complementation construct was transformed into the Δ*MaCsbD* mutant using the *Agrobacterium*-mediated method. Complemented strains (CP) were selected on Czapek-Dox medium containing 60 µg/mL chlorsulfuron ethyl (Sigma, Bellefonte, PA, USA) [21]. Genotypic validation was performed through PCR screening, followed by quantitative reverse transcription PCR (qRT-PCR) to assess transcriptional restoration (Figure S1).

### 2.4. Conidial Germination and Conidiation Capacity Analysis

Conidial germination assays were performed as previously described [22]. Conidial suspensions (1 × 10^7^ conidia/mL) of fungal strains were prepared with sterile 0.05% Tween-80. Then, 100 μL was evenly coated on the medium plate and cultured at 28 °C. At 2 h intervals for up to 12 h, plates were removed. A 1 cm^2^ section of agar was excised from each plate, mounted on a glass slide, and examined under a light microscope. The number of germinated conidia was determined by counting at least 100 conidia per sample. Germination was defined by the emergence of a germ tube longer than half the conidial diameter. Each strain was assayed in triplicate plates per experiment, and the experiment was independently repeated three times. Conidial production was determined as described in Methods. Conidial suspensions of WT, ∆*MaCsbD*, and CP (1 × 10^6^ conidia/mL) were added to 24-well plates containing 2 mL of medium and incubated continuously at 28 °C for 15 days. Every 3 days, conidia were harvested by adding 1 mL of sterile 0.05% (*v*/*v*) Tween-80 solution to each well and gently scraping the culture surface with a sterile pipette tip. The resulting suspension was collected, and conidial concentration was determined using a hemocytometer [23]. Each group was counted in triplicate, and the experiment was repeated three times.

### 2.5. Stress Resistance Analysis

Fungal tolerance to UV-B irradiation and heat shock was determined as described previously [24]. Briefly, conidial suspensions (1 × 10^7^ conidia/mL) of the WT, Δ*MaCsbD*, and CP strains were prepared. Aliquots (100 μL) of each suspension were evenly spread onto 1/4 SDAY plates. For UV-B tolerance assays, plates were exposed to UV-B radiation (1350 mW/m^2^) for 1, 2, 3, 4, or 5 h [25]. For heat shock tolerance assays, plates were incubated at 45 °C for 2, 4, 6, 8, 10, or 12 h. Following stress treatment, all plates were incubated at 28 °C for 20 h. Conidial germination rates were then determined microscopically as described in Section 2.4. Fungal tolerance to hyperosmotic and oxidative stress, as well as susceptibility to cell wall stressors, was assessed on 1/4 SDAY plates supplemented with 0.1 M NaCl, 1 M sorbitol (SOR), 6 mM H_2_O_2_, 0.01% (*w*/*v*) SDS, 50 µg/mL calcofluor white (CFW), or 500 µg/mL Congo red (CR). WT, ∆*MaCsbD* and CP strains were detected on 1/4 SDAY in the absence and presence of stressors. Plates were incubated at 28 °C for 6 days. Fungal colonies were photographed, and colony growth rates were measured by determining the average colony diameter.

### 2.6. Trehalose Extraction and Quantification

Trehalose was extracted according to the instructions provided by the trehalose content determination kit (Beijing Solarbio Science & Technology Co., Ltd., Beijing, China) [26]. Three replicates were set for each strain, and the extracted trehalose was quantitatively analyzed. 0.1 g of *M. acridum* (WT, Δ*MaCsbD*, CP) cultured for 15 days was taken, and extracted with 1 mL of trichloroacetic acid solution. At 4 °C, the fungal conidia were disrupted by ultrasonic treatment (200 W, 3 s on, 10 s off, repeated 30 times). The mixture was left to stand at room temperature for 45 min, shaken 3–5 times, and then centrifuged at 8000 rpm for 10 min at room temperature to obtain the supernatant. Then, 250 μL of the supernatant of each sample was transferred to an EP tube, and 1 mL of anthrone-sulfuric acid reagent was added. The reaction mixture was incubated in a 95 °C water bath for 10 min. After cooling to room temperature, the absorbance (A) was measured at 620 nm. The trehalose standard was diluted with distilled water to 0.1, 0.05, 0.025, 0.0125, 0.00625, 0.003125, and 0 mg/mL, and the corresponding absorbance values were measured to draw the standard curve. The trehalose content was calculated based on the dry weight of the conidia, and the experiment was repeated three times.

### 2.7. Determination of Melanin Content

Melanin was extracted from *M*. *acridum* conidia using a modified protocol based on Qin [27]. Three biological replicates were prepared for each strain (WT, Δ*MaCsbD*, CP). Conidial suspensions (1 × 10^8^ conidia/mL) were prepared in 1 mol/L NaOH. Pigment extraction was performed by boiling suspensions in a 100 °C water bath for 120 min. After cooling, samples were centrifuged at 12,000 rpm/min for 10 min. The supernatant was filtered through a 0.22 μm microporous membrane. Melanin content was quantified spectrophotometrically at 485 nm. A standard curve was generated using serial dilutions of synthetic melanin (Sigma-Aldrich, St. Louis, MO, USA) dissolved in 1 mol/L NaOH. All extractions and measurements were performed in three independent experimental replicates.

### 2.8. ROS Scavenging Capacity Analysis

According to the method described by Song et al., *M*. *acridum* was prepared into a conidial suspension with a final concentration of 1 × 10^7^ conidia/mL. [28]. Each conidial suspension (100 µL) was plated onto 1/4 SDAY plates and exposed to UV-B light at 1350 mW/m^2^ for four hours or moist heat treatment for 10 h. The total intracellular reactive oxygen species (ROS) levels in conidia from each strain were quantified using 2′,7′-dichlorodihydrofluorescein diacetate (DCFH-DA) (Solarbio, Beijing, China). The fluorescence signal generated by the oxidized probe (DCF) was visualized and captured using a confocal laser scanning microscope (Nikon, Tokyo, Japan). Following UV-B treatment, conidia were incubated at 28 °C for 20 h to allow for recovery. Subsequently, DCFH-DA staining and fluorescence imaging were performed as described above. The enzymatic activities of key reactive oxygen species scavenging enzymes—catalase (CAT), superoxide dismutase (SOD), glutathione peroxidase (GPX), and peroxidase (POD)—were assayed according to the manufacturers’ instructions using commercial kits (Solarbio, Beijing, China; Beyotime, Shanghai, China) [29]. All experiments were performed with three independent biological replicates, and the entire experimental series was repeated three times.

### 2.9. DNA Damage Repair Assessment

Following the experimental protocol described in Qin, conidial suspensions of the WT, Δ*MaCsbD*, and CP strains were prepared by dilution in 0.05% (*v*/*v*) Tween-80 to a final concentration of 1 × 10^7^ conidia/mL. Each conidial suspension (100 µL) was plated onto 1/4 SDAY plates and exposed to UV-B light at 1350 mW/m^2^ for 4 h or heat shock treatment for 10 h. Three biological replicates per sample underwent identical therapies to those in the ROS assay. Following treatments, conidia were stained with 4′,6-diamidino-2-phenylindole (DAPI; Beyotime, Shanghai, China). Nuclear morphology was visualized and imaged using a laser scanning confocal microscope (LSCM). UV-B or heat-treated conidia were subsequently incubated at 28 °C for 20 h before DAPI staining and imaging as described. Three biological replicates per strain per condition were analyzed. The entire experimental series was independently repeated three times.

### 2.10. Virulence Analysis

*Locusta migratoria manilensis* were kept at 30 °C, 75% relative humidity, and 12 h L:12 h D light. The conidia of *M. acridum* cultured for 15 days were prepared with liquid paraffin at a final concentration of 1 × 10^7^ conidia/mL for virulence analysis as described previously [30]. For spot virulence analysis, 5 μL of the conidial suspension was sprinkled onto the dorsal plate of the fifth instar larvae of East Asian locusts, and 5 μL of liquid paraffin was used as the blank control. Thirty-fifth-instar locusts were placed in one cage, and a total of three cages were used for biological testing. After the treatment, feed the locusts with corn leaves and count the number of their deaths every 0.5 days.

### 2.11. qRT-PCR

Total RNA was extracted from conidia using an Ultrapure RNA Kit (with DNase I) (CWBIO, Beijing, China). The RNA samples were then reverse-transcribed utilizing the PrimeScript RT Reagent Kit with gDNA Eraser (UE, Tianjin, China), following the manufacturer’s instructions. Gene-specific qRT-PCR primers were designed utilizing the NCBI website (https://www.ncbi.nlm.nih.gov/; accessed on 2 March 2025). The qRT-PCR was conducted using the SYBR Prime qRT-PCR Set (Bio Ground, Chongqing, China) following a two-step method in accordance with the manufacturer’s protocol [31]. The transcriptional levels of target genes were calculated using the 2−∆∆Ct method. The glycerol-3-phosphate dehydrogenase gene (GenBank accession number: EFY84384) was used as the reference gene. Each reaction was carried out in triplicate. The sequences of all primers used for qRT-PCR in this study are provided in Supplementary Table S1.

### 2.12. Data Analyses

The primers were designed using Primer Premier v 5.0 software. Statistical analysis of the experimental data was conducted using one-way analysis of variance (ANOVA) in SPSS 16.0. Graphpad Prism v 5.0 software and Adobe Photoshop 2022 software were used for image processing. The Tukey test (using SPSS 16.0 software) was employed to determine the differences in the mean values (±standard error).

## 3. Results

### 3.1. Phylogenetic and Structural Analysis Reveals MaCsbD as a Conserved Fungal Protein

**Figure 1:**
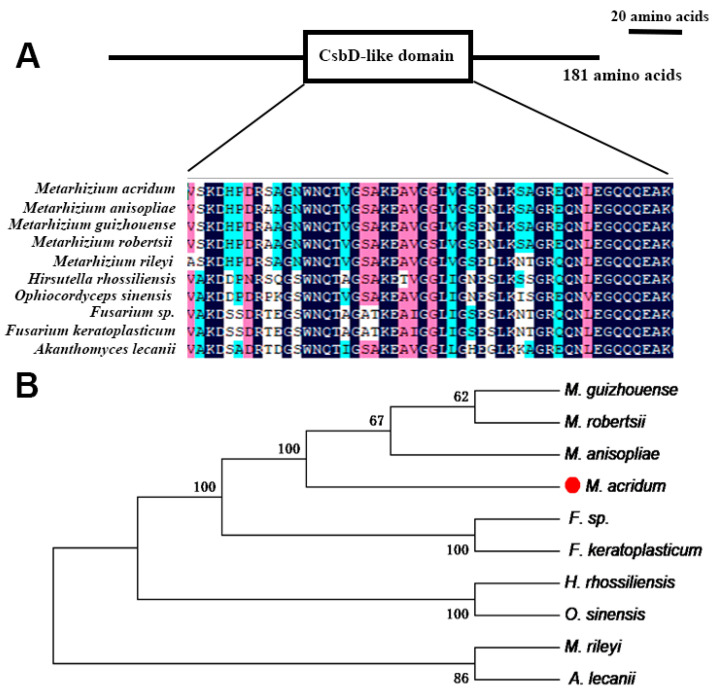
Features of *MaCsbD*. (**A**) The location and multiple sequence alignment analysis of CsbD-like domain. The sequences are colored based on sequence conservation at each position, with darker shades indicating higher conservation. (**B**) Phylogenetic analysis of CsbD-like proteins across diverse fungal species was conducted using MEGA software. The red dot highlights the position of the species under primary investigation in this study, *Metarhizium acridum*. The numbers at the branch nodes represent bootstrap values based on 1000 replicates. Species names are abbreviated as follows: *Metarhizium acridum* (*M. acridum*), *Metarhizium anisopliae* (*M. anisopliae*), *Metarhizium robertsii* (*M. robertsii*), *Metarhizium guizhouense* (*M. guizhouense*), *Metarhizium rileyi* (*M. rileyi*), *Hirsutella rhossiliensis* (*H. rhossiliensis*), *Ophiocordyceps sinensis* (*O. sinensis*), *Fusarium sp*. (*F. sp*.), *Fusarium keratoplasticum* (*F. keratoplasticum*), and *Akanthomyces lecanii* (*A. lecanii*).

The *MaCsbD* gene, with a 619 bp coding sequence, was identified by NCBI with the accession number J3458_004571 and the protein accession number XP_007807437.1. *MaCsbD* was predicted to encode a 181 amino acid protein. Expasy analysis showed that the molecular weight of the protein was 18.56 kDa, and the isoelectric point was 5.25. Domain prediction of *MaCsbD* using SMART showed that it contains a CsbD-like domain (Pfam: pfam05532). Domain prediction indicates that the CsbD domain is highly conserved in fungi (Figure 1A). A phylogenetic tree was constructed based on a multiple sequence alignment of CsbD and its homologs from various fungi. The resulting phylogeny demonstrates that *MaCsbD* clusters most closely with the protein from *Metarhizium anisopliae*.

### 3.2. MaCsbD Is Required for Efficient Conidial Germination and Production

**Figure 2:**
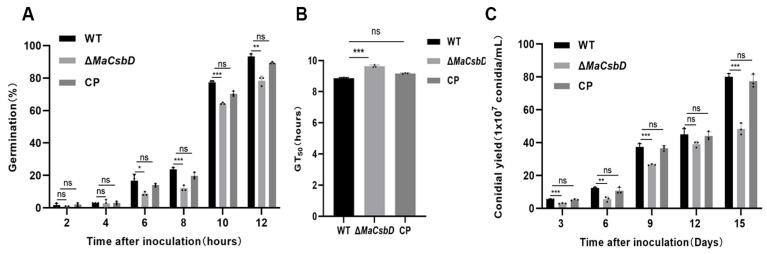
*MaCsbD* is required for normal conidial germination and yield. (**A**) Time-course of conidial germination rates for the Wild-Type (WT), Δ*MaCsbD* knockout mutant, and Complemented Strain (CP) on 1/4 SDAY medium. (**B**) The mean time required for 50% of conidia to germinate (GT_50_). (**C**) Conidial production over 15 days of culture. Error bars represent the mean ± SEM (*n* = 3). Asterisks indicate significant differences (* *p* < 0.05; ** *p* < 0.01; *** *p* < 0.001; ns, not significant) as determined by one-way ANOVA with Tukey’s test.

We performed a functional analysis of the *MaCsbD* gene to determine its importance for conidial germination and production in *M. acridum*. Conidial germination and yield of strains WT, Δ*MaCsbD*, and CP were measured on 1/4 SDAY medium. The conidial germination rate of Δ*MaCsbD* was significantly lower than that of WT strain (Figure 2A). The germination rate of conidia in WT strain was approximately 30% after 8 h of culture, while the germination rate of Δ*MaCsbD* strain was less than 15%. The mean 50% germination time (GT_50_) of Δ*MaCsbD* was significantly higher than that of WT and CP strains (Figure 2B). To investigate the effect of Δ*MaCsbD* on the conidial production of *M. acridum*, the amount of conidiation of Δ*MaCsbD* was measured. By counting the germination rates of Δ*MaCsbD* and WT, it was found that from the 3rd day of culture of Δ*MaCsbD*, the conidial production of WT was significantly higher than that of Δ*MaCsbD* strain (Figure 2C).

### 3.3. MaCsbD Is Essential for Tolerance to UV-B Irradiation and Heat Shock

**Figure 3:**
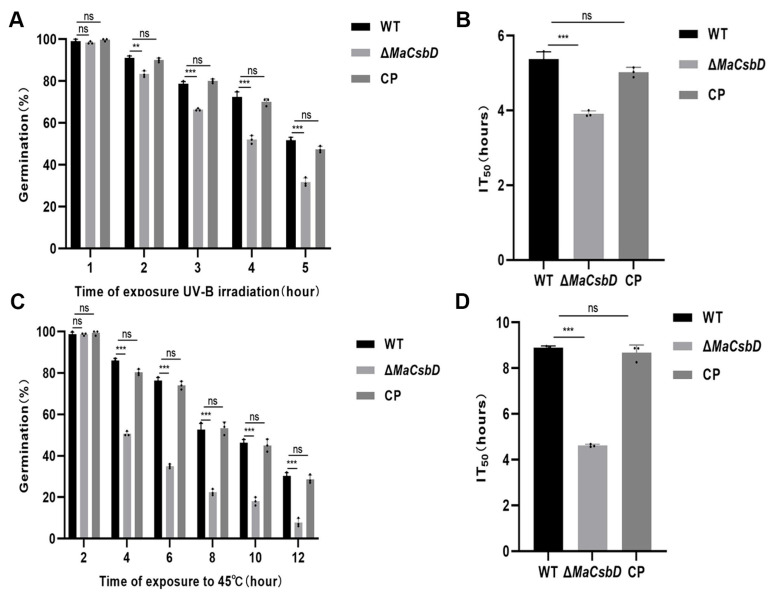
*MaCsbD* is essential for tolerance to UV-B and heat shock. (**A**) Germination rates of strains after exposure to UV-B irradiation (1350 mW/m^2^) for the indicated durations. (**B**) Median inhibition time (IT_50_, the irradiation time causing 50% germination inhibition). (**C**) Germination rates after heat shock (45 °C) for the indicated durations. (**D**) IT_50_ for heat shock. Data are presented as mean ± SEM (*n* = 3). ** *p* < 0.01; *** *p* < 0.001; ns, not significant.

To investigate the growth of Δ*MaCsbD* under stress conditions, the germination rate of Δ*MaCsbD* was measured under wet heat and UV-B conditions. After treatment with UV-B for 1, 2, 3, 4, and 5 h, the germination rate of Δ*MaCsbD* decreased to some extent compared with that of WT (Figure 3A). The results showed that 50% inhibition time (IT_50_) values of the WT, Δ*MaCsbD*, and CP strains were 5.36, 3.91, and 5.02, respectively. The value of the Δ*MaCsbD* strain was significantly lower than that of the WT and CP strains (*p* < 0.001) (Figure 3B). Similarly, after being treated under heat shock conditions of 45 °C (Figure 3C), the germination rate of Δ*MaCsbD* was significantly lower than that of WT and CP. The IT_50_ values of WT, Δ*MaCsbD*, and CP strains were 8.89, 4.61, and 8.69, respectively. Δ*MaCsbD* was significantly lower than both WT and CP (*p* < 0.001) (Figure 3D).

### 3.4. MaCsbD Confers Resistance to Osmotic and Cell Wall Stressors

**Figure 4:**
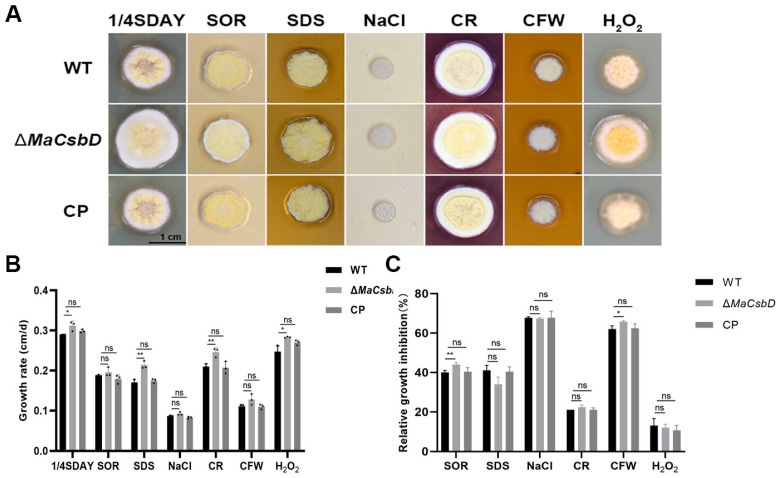
Sensitivity of Δ*MaCsbD* to chemical stressors. (**A**) Colony morphology of WT, Δ*MaCsbD*, and CP strains on 1/4 SDAY amended with 1 M sorbitol (SOR), 0.01% SDS, 0.1 M NaCl, 500 µg/mL Congo red (CR), 50 µg/mL calcofluor white (CFW), or 6 mM H_2_O_2_ after 6 days. (**B**) Colony growth rates. (**C**) Relative Growth Inhibition (RGI) under stress conditions. Data are mean ± SEM (*n* = 3). * *p* < 0.05; ** *p* < 0.01; ns, not significant.

To determine the effects of different chemicals on Δ*MaCsbD* strain, the conidia of WT, Δ*MaCsbD*, and CP strains were cultured in 1/4 SDAY medium supplemented with hypertonic substances (sorbitol, NaCl), oxygen stress substances (H_2_O_2_), cell wall destruction, and SDS or Congo red (CR). The results showed that the colony size of Δ*MaCsbD* strain was significantly larger than that of WT strain on 1/4 SDAY medium. The colony size of Δ*MaCsbD* strain was slightly larger than that of WT strain on the medium supplemented with NaCl, H_2_O_2_, CR, and SDS (Figure 4A,B). These results indicated that the *MaCsbD* knockout strain exhibits significantly reduced tolerance to NaCl, H_2_O_2_, CR, and SDS. Δ*MaCsbD* had 43.7% Relative Growth Inhibition (RGI) when SOR was included in the medium, significantly higher than WT and CP strains (*p* < 0.01), which were 40.2% and 41.3%, respectively. Similarly, Δ*MaCsbD* had 66.1% RGI when calcofluor white (CFW) was included in the medium, higher than WT and CP strains (*p* < 0.05), which were 60.9% and 61.2%. However, the RGI of Δ*MaCsbD* had no significant difference with WT and CP under SDS, NaCl, CR and H_2_O_2_ stressors (Figure 4C).

### 3.5. MaCsbD Positively Regulates Melanin Biosynthesis

**Figure 5:**
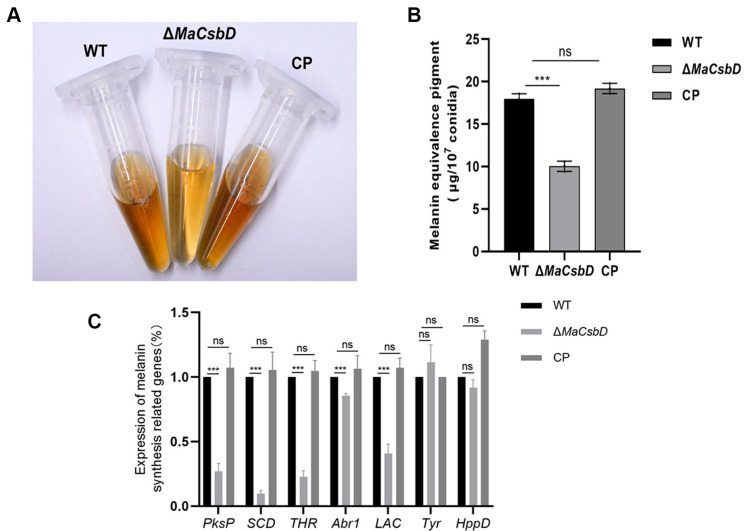
Deletion of *MaCsbD* impairs melanin biosynthesis. (**A**) Visual comparison of melanin pigmentation in conidia from 15-day-old cultures of WT, Δ*MaCsbD*, and CP strains. (**B**) Quantitative spectrophotometric analysis of melanin content extracted from conidia. (**C**) Relative transcriptional levels of key genes in the DHN-melanin pathway—polyketide synthase (*PksP*), T4HN reductase (*THR*), and scytalone dehydratase (*SCD*)—as determined by qRT-PCR. The glyceraldehyde-3-phosphate dehydrogenase gene (*GPDH*) was used as an internal reference. Data are mean ± SEM (*n* = 3). Significant differences were determined by one-way ANOVA with Tukey’s test. *** *p* < 0.001; ns, not significant.

Melanin is a multifunctional protective molecule. It can neutralize reactive oxygen species and absorb ultraviolet radiation, thereby protecting enzymes and cell membranes under environmental stress. To elucidate the molecular effects of *MaCsbD* on the heat resistance and ultraviolet radiation tolerance of the *M. acridum*, we extracted melanin from mature conidia of the WT, Δ*MaCsbD*, and CP strains grown on 1/4 SDAY medium plates for 15 days (Figure 5A) and conducted quantitative analysis. The results showed that the melanin content in the conidia of Δ*MaCsbD* was significantly lower than that in the conidia of WT and CP (Figure 5B). We also extracted RNA from the three strains to quantitatively analyze the transcriptional expression of key genes in the fungal melanin synthesis pathway. The RT-qPCR results indicated that the knockout of *MaCsbD* led to a significant downregulation of the transcriptional expression of genes encoding the rate-limiting enzymes in the DHN-melanin synthesis pathway, including polyketide synthase(*PksP*), T4HN reductase (*THR*), and Scytalone dehydratase,(*SCD*) (Figure 5C). This suggests that the deletion of the *MaCsbD* gene would hinder melanin synthesis. The reduction in heat-shock and ultraviolet radiation tolerance caused by the deletion of *MaCsbD* may be related to the decrease in melanin synthesis.

### 3.6. MaCsbD Mediates Thermotolerance Through Enhanced DNA Repair, HSP Induction, ROS Clearance, and Trehalose Accumulation in Conidia

**Figure 6:**
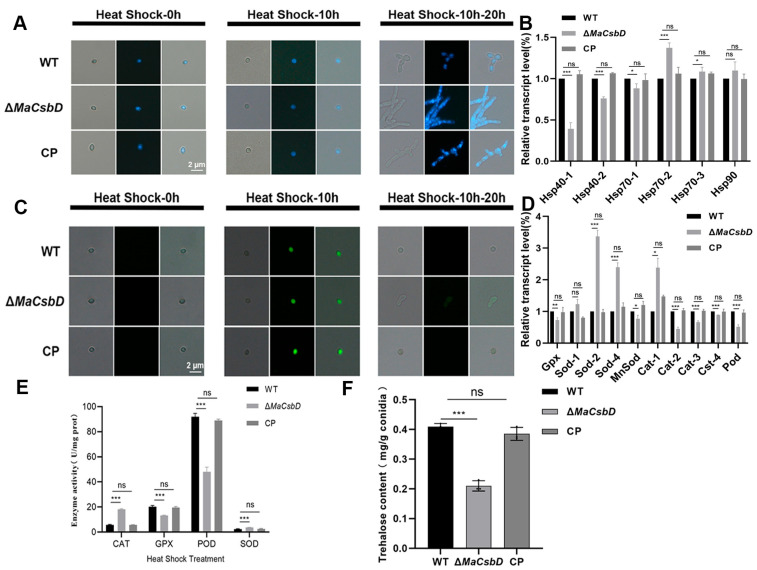
*MaCsbD* mediates thermotolerance through enhanced DNA repair, HSP induction, and ROS clearance, and is required for trehalose accumulation. (**A**) Nuclear DNA morphology assessed by DAPI staining. “Heat Shock-0 h”: no treatment; “Heat Shock-10 h”: after 10 h at 45 °C; “Heat Shock-10–20 h”: after 10 h heat shock followed by 20 h recovery at 28 °C. White arrows indicate dispersed nuclei indicative of DNA damage. (**B**) Relative expression of heat shock protein genes (*Hsp40-1*, *Hsp40-2*, *Hsp70-1*) after heat shock, analyzed by qRT-PCR. (**C**) Intracellular ROS levels detected by DCFH-DA staining (green fluorescence). The treatment timeline is identical to (**A**). (**D**) Relative expression of antioxidant enzyme genes (*Gpx*, *MnSod*, *Cat2*, *Cat3*, *Cat4*, *Pod*) after heat shock. (**E**) Enzymatic activities of catalase (CAT), glutathione peroxidase (GPX), peroxidase (POD), and superoxide dismutase (SOD) after heat shock treatment. (**F**) Trehalose content in conidia from 15-day-old cultures. Data are mean ± SEM (*n* = 3). Significant differences were determined by one-way ANOVA with Tukey’s test. * *p* < 0.05; ** *p* < 0.01; *** *p* < 0.001; ns, not significant.

To investigate the regulatory role of *MaCsbD* in heat shock, we analyzed DNA repair and reactive oxygen species (ROS) dynamics in *M. acridum* strains (WT, Δ*MaCsbD*, and CP) following a 10 h heat-shock stress and a subsequent 20 h recovery period. DAPI staining revealed a diffuse nuclear morphology in all strains post-treatment, indicating DNA damage. After recovery, conidia from WT and CP strains exhibited re-aggregated nuclei, whereas Δ*MaCsbD* nuclei remained dispersed. This demonstrates impaired DNA repair capacity in the mutant (Figure 6A). RT-qPCR further confirmed the downregulation of heat shock protein (HSP) genes (*Hsp40-1*, *Hsp40-2*, and *Hsp70-1*) in the Δ*MaCsbD* strain (Figure 6B). Concurrently, ROS clearance was assessed via DCFH-DA staining. Post-treatment and recovery, WT and CP conidia completely cleared ROS (no fluorescence). At the same time, Δ*MaCsbD* exhibited persistent fluorescence (Figure 6C). This correlated with transcriptional downregulation of antioxidant genes (*Gpx*, *MnSod*, *Cat2*, *Cat3*, *Cat4*, *Pod*) in Δ*MaCsbD*, highlighting its attenuated ROS detoxification capacity mediated by redox enzyme systems (Figure 6D). The activities of CAT, GPX, POD, and SOD were measured in the WT, Δ*MaCsbD*, and CP strains after heat shock treatment. The results showed that the activities of GPX and POD were significantly decreased (Figure 6E).

*MaCsbD* acts as a positive regulator of heat shock stress response. Its deletion compromises tolerance through the following mechanisms: (1) Inhibition of HSP synthesis, promoting protein denaturation; (2) reduced ROS scavenging capability via downregulation of antioxidant gene expression; (3) Impaired DNA repair. These findings establish *MaCsbD* as a key facilitator in stress adaptation pathways. Its gene knockout sensitizes the organism to heat shock stress.

Trehalose is present in fungal cell walls and plays a critical role in maintaining structural stability, mediating environmental stress responses, and regulating pathogenicity. Under heat, drought, or hypertonic stress, trehalose mitigates depolymerization or rearrangement of cell wall polysaccharides, thereby preserving the integrity of the three-dimensional network structure and protecting cells. To elucidate the molecular effects of *MaCsbD* on heat tolerance and UV-B radiation tolerance in *M. acridum*, trehalose was extracted from mature conidia of WT, Δ*MaCsbD*, and CP strains cultured on 1/4 SDAY agar plates for 15 days, followed by quantitative analysis. The results demonstrated that trehalose content in Δ*MaCsbD* conidia was significantly lower than in WT and CP conidia (Figure 6F). This indicates that *MaCsbD* knockout impairs trehalose synthesis, and the observed reductions in moist heat tolerance and UV-B radiation tolerance resulting from *MaCsbD* deletion may be associated with diminished trehalose accumulation.

### 3.7. MaCsbD Promotes UV-B Resistance by Facilitating DNA Repair and Antioxidant Defense

**Figure 7:**
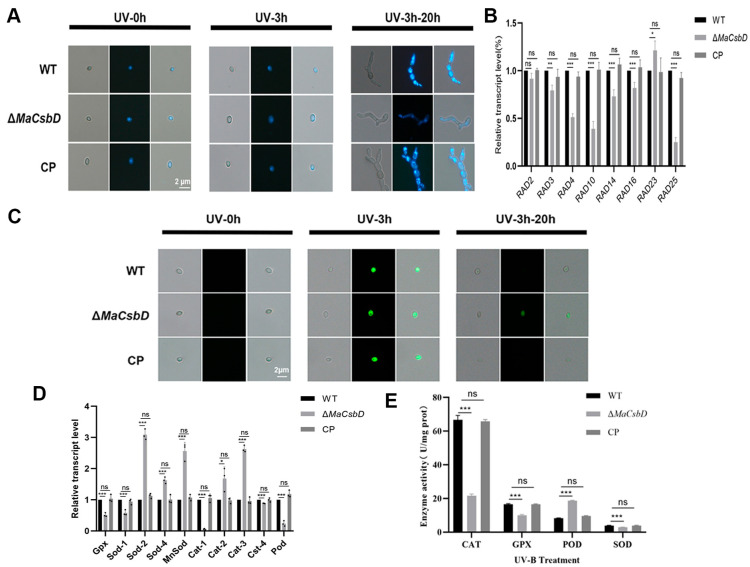
*MaCsbD* promotes UV-B resistance by facilitating DNA repair and antioxidant defense. (**A**) Nuclear DNA morphology assessed by DAPI staining. “UV-0 h”: no treatment; “UV-3 h”: after 3 h of UV-B irradiation; “UV-3–20 h”: after 3 h UV-B followed by 20 h recovery at 28 °C. White arrows indicate persistent nuclear dispersion. (**B**) Relative expression of nucleotide excision repair (NER) genes (*Rad2*, *Rad3*, *Rad4*, *Rad10*, *Rad14*, *Rad16*, *Rad25*) after UV-B irradiation. (**C**) Intracellular ROS levels detected by DCFH-DA staining (green fluorescence). The treatment timeline is identical to (**A**). (**D**) Relative expression of antioxidant enzyme genes (*Gpx*, *Sod1*, *Cat1*, *Cat4*, *Pod*) after UV-B irradiation. (**E**) Enzymatic activities of CAT, GPX, POD, and SOD after UV-B treatment. Data are mean ± SEM (*n* = 3). Significant differences were determined by one-way ANOVA with Tukey’s test. * *p* < 0.05; ** *p* < 0.01; *** *p* < 0.001; ns, not significant.

To clarify the regulatory role of *MaCsbD* in UV-B tolerance, we analyzed DNA repair capacity and reactive oxygen species (ROS) scavenging capability in *M. acridum* strains (WT, Δ*MaCsbD*, and CP). DAPI staining revealed that UV-B treatment (3 h) induced a diffuse nuclear morphology in conidia of all strains, indicating severe DNA damage. Following a 20 h recovery period, Δ*MaCsbD* conidia retained dispersed nuclei. In contrast, WT and CP strains exhibited nuclear recondensation, demonstrating impaired DNA repair capacity upon *MaCsbD* knockout (Figure 7A). RT-qPCR analysis further showed downregulation of nucleotide excision repair (NER) pathway genes (*Rad2, Rad3, Rad4, Rad10, Rad14, Rad16, Rad25*) in Δ*MaCsbD*, confirming compromised NER functionality (Figure 7B). Concurrently, ROS dynamics assessed via DCFH-DA staining revealed complete ROS clearance (absence of fluorescence) in UV-B treated WT and CP conidia after 20 h. In contrast, Δ*MaCsbD* conidia exhibited persistent weak green fluorescence (Figure 7C). This correlated with transcriptional downregulation of antioxidant enzymes (*Gpx*, *Sod1*, *Cat1*, *Cat4*, *Pod*) in the mutant (Figure 7D), highlighting its attenuated ROS detoxification capacity mediated by the POD/CAT/SOD/GPX enzymatic system. The activities of CAT, GPX, POD, and SOD were measured in the WT, Δ*MaCsbD*, and CP strains after UV-B treatment. The results showed that the activities of CAT, GPX, and SOD were significantly decreased (Figure 7E). *MaCsbD* positively regulates the UV-B-induced stress response in *M. acridum*. Its deletion compromises UV-B resistance through: (1) Attenuation of NER-mediated DNA repair, (2) Reduced ROS scavenging capability via downregulation of antioxidant gene expression, and (3) Impaired nuclear recovery. These findings establish *MaCsbD* as a key facilitator in stress adaptation pathways, providing novel insights into the molecular mechanisms underlying fungal UV-B tolerance

### 3.8. MaCsbD Deletion Does Not Impair Fungal Virulence

**Figure 8:**
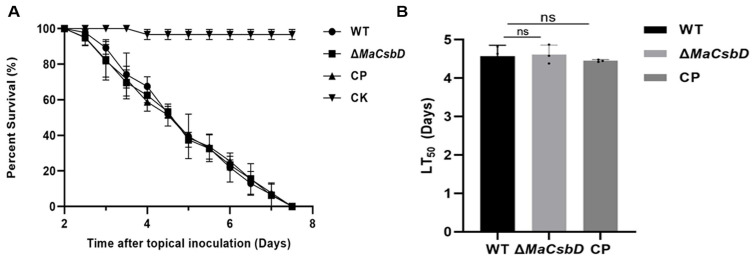
*MaCsbD* deletion does not impair fungal virulence against locusts. (**A**) Survival curves of fifth-instar *Locusta migratoria manilensis* nymphs after topical inoculation with 5 μL of conidial suspension (1 × 10^7^ conidia/mL) of WT, Δ*MaCsbD*, CP and CK (Control Check) strains. Liquid paraffin was used as a blank control. (**B**) The median lethal time (LT_50_) for each strain. Error bars indicate the standard error of the mean. No significant differences (ns, *p* > 0.05) were found as determined by one-way ANOVA with Tukey’s test.

*M. acridum* is a critical entomopathogenic fungus whose virulence against locusts directly determines the efficacy of mycoinsecticidal formulations. To investigate the role of *MaCsbD* in fungal pathogenicity, fifth-instar nymphs of the *Locusta migratoria manilensis* were subjected to topical inoculation assays to evaluate the in vivo virulence of WT, Δ*MaCsbD*, and CP strains. Bioassays demonstrated that locusts inoculated with either WT, Δ*MaCsbD*, and CP strains exhibited less than 50% survival by day 5 post-inoculation, with complete mortality achieved by day 8 (Figure 8A). Comparative analysis revealed no significant differences in virulence between the Δ*MaCsbD* mutant and the WT or complemented strain (CP), indicating that *MaCsbD* deletion does not impair fungal pathogenicity under these experimental conditions (Figure 8B).

## 4. Discussion

The biological functions of CsbD-like proteins have been extensively explored in prokaryotes. Yet, their roles in eukaryotes remained completely unknown. Our study provides the first functional evidence that a CsbD homolog contributes to stress tolerance in fungi. Specifically, we demonstrate that *MaCsbD* in the entomopathogenic fungus *M. acridum* supports thermotolerance, UV-B survival, and conidiation. Compared with the WT and complemented strains, the Δ*MaCsbD* mutant exhibited a 47% reduction in melanin accumulation and a 50% reduction in trehalose content, delayed germination, and decreased conidial yield. After UV-B irradiation and heat shock treatment, the IT_50_ values of the conidia decreased by 1.45 and 4.28 days, respectively. These defects coincided with significantly impaired tolerance to UV-B irradiation and heat shock, while pathogenicity toward locust hosts remained unaffected. These findings not only reveal a functional divergence from the bile salt-related mechanisms of bacterial CsbD proteins but also identify a previously uncharacterized regulatory component in fungal stress adaptation.

Mechanistically, our data position *MaCsbD* as a node influencing multiple cellular pathways that collectively determine fungal resistance to environmental challenges. Our data show that deletion of *MaCsbD* downregulated the expression of *Hsp40* and *Hsp70* family genes, resulting in a weakened heat shock response. Heat shock proteins are central molecular chaperones that assist in protein folding, repair misfolded polypeptides, and maintain proteostasis during thermal stress. In *S. cerevisiae*, *Hsp104* accumulation is well known to enhance tolerance to elevated temperatures [32,33]. The downregulation of HSP expression in Δ*MaCsbD* thus provides a mechanistic explanation for the marked decrease in thermotolerance observed in our assays.

UV-B sensitivity of the mutant can be similarly explained by impaired DNA repair capacity. UV-B irradiation causes characteristic DNA lesions such as cyclobutane pyrimidine dimers (CPDs) and 6-4 photoproducts [34]. While fungi possess both photorepair and nucleotide excision repair (NER) pathways to mitigate such lesions, NER often represents the dominant defense [35,36]. In *Aspergillus*, NER genes are strongly induced after UV-B exposure [37], and in *M. robertsii*, *Rad2*, *Rad14*, and *Rad26* homologs have been linked to conidial UV-B resistance [13]. In the Δ*MaCsbD* mutant, the downregulation of multiple NER-related genes, including *Rad2*, *Rad3*, *Rad14*, and *Rad26*, suggests that *MaCsbD* is required for full induction of this DNA repair system. The loss of this capacity likely explains the accumulation of DNA damage and the markedly reduced survival of Δ*MaCsbD* conidia following UV-B exposure.

Beyond HSPs and DNA repair, *MaCsbD* also influences protective metabolite synthesis. In the mutant, melanin and trehalose were both significantly reduced. Melanin, a protective pigment, helps neutralize ROS and absorb UV-B radiation, which likely explains the increased stress sensitivity of the mutant. Melanin is well known to absorb UV-B radiation, neutralize reactive oxygen species (ROS), and provide structural defense for fungal cell walls [38]. Its depletion in Δ*MaCsbD* is consistent with the higher UV-B sensitivity we observed. This phenotype resembles previous reports in *M. anisopliae*, where melanin-deficient mutants exhibited impaired ROS clearance and decreased UV-B survival. Trehalose, on the other hand, functions as a chemical chaperone that stabilizes proteins and cell membranes under desiccation and thermal stress. The 50% reduction in trehalose levels in Δ*MaCsbD* directly correlates with sensitivity to heat shock, as the loss of this sugar disrupts osmotic balance and weakens resistance to protein denaturation [39]. Together, reduced melanin and trehalose levels compromise both physical and chemical barriers to environmental stress, magnifying the defects of the *MaCsbD* mutant.

ROS homeostasis also appears to be affected. Following UV-B or moist heat treatments, the Δ*MaCsbD* strain exhibited significantly decreased ROS-scavenging ability. This may be a downstream consequence of impaired melanin and trehalose synthesis, but could also reflect broader disruption of antioxidant enzyme networks. The concurrent downregulation of antioxidant enzyme genes (*Gpx, MnSod, Cat2*, etc.; Figure 6D and Figure 7D) in Δ*MaCsbD* suggests that this impaired ROS clearance is also a direct consequence of a disrupted transcriptional regulatory network controlled by *MaCsbD*. In fungi, ROS detoxification is closely linked to the activities of catalase, superoxide dismutase, and glutathione peroxidase. Downregulation of these genes in Δ*MaCsbD* further supports the conclusion that *MaCsbD* orchestrates multiple arms of the oxidative stress defense system.

Significantly, despite these pronounced defects, *MaCsbD* deletion did not attenuate fungal virulence. This distinguishes it from other stress-related regulators such as *MaHog1* [18] or *MaAreB* [32], where loss of stress tolerance is often accompanied by reduced pathogenicity. The apparent decoupling of stress resistance and virulence in *MaCsbD* is significant for applied research: it suggests that stress-tolerant strains could be engineered without compromising infectivity. Phylogenetic analysis further revealed that CsbD-like proteins are conserved among entomopathogenic fungi, especially within the *Metarhizium* genus, suggesting an evolutionarily conserved role in abiotic stress adaptation. Interestingly, this function diverges from that of bacterial homologs, which are primarily involved in bile salt resistance, pointing to lineage-specific specialization of CsbD-like proteins across kingdoms.

Overall, our findings point to *MaCsbD* as a key regulator of stress adaptation, with practical implications for improving fungal biocontrol. By integrating HSP-mediated proteostasis, NER-dependent DNA repair, ROS detoxification, and metabolite-based defenses, *MaCsbD* provides *M. acridum* with resilience to UV-B radiation and heat stress while preserving pathogenicity.

From an applied perspective, the specific role of *MaCsbD* in abiotic stress adaptation, uncoupled from virulence, makes it a promising molecular target for improving the performance of fungal biocontrol agents. Enhancing its expression could increase conidial production, extend shelf life, and boost field persistence—traits that remain limiting factors in large-scale agricultural use. Unlike broader stress regulators that interfere with virulence pathways, *MaCsbD*’s selective role in abiotic stress adaptation makes it particularly attractive for strain improvement.

Although our results establish the importance of *MaCsbD*, its precise regulatory mechanisms remain unclear. Future work should aim to identify the upstream regulators that control *MaCsbD* expression, map its protein interaction partners, and test whether it directly binds to the promoters of stress-related genes using approaches such as ChIP-seq. Proteomic analyses could reveal additional pathways under *MaCsbD* control, while engineered overexpression lines would allow for assessment of its potential in strain improvement. Ultimately, these studies will deepen our understanding of fungal stress adaptation and help validate *MaCsbD* as a molecular handle for developing stress-tolerant, high-performance fungal biopesticides under field conditions.

## 5. Conclusions

In summary, *MaCsbD* plays a pivotal role in key stress tolerance traits and growth characteristics of *M. acridum*, including conidial yield and development, and resistance to abiotic stressors. Our research not only reveals the basic function of *MaCsbD* in *M. acridum*, but also clarifies the molecular mechanism regulating resistance to UV-B irradiation and heat shock. Therefore, this study provides important insights and a genetic foundation for strategies aimed at improving the environmental resilience and biocontrol efficacy of fungal insecticides.

## Data Availability

The original contributions presented in this study are included in the article/Supplementary Materials. Further inquiries can be directed to the corresponding author.

## Supplementary Materials

The following supporting information can be downloaded at: https://www.mdpi.com/article/10.3390/jof11120838/s1, Figure S1: The disruption and complementation of *MaCsbD*. (**A**) The knockout and complement schematic diagram of *MaCsbD*. (**B**) PCR was used to verify the complementary strains of *MaCsbD*. (**C**) Transcription-level analysis of *MaCsbD* in WT, Δ*MaCsbD*, and CP strains. *MaCsbD* expression in WT as control. Asterisks indicate significant differences (*** *p* < 0.001; ns, not significant) as determined by one-way ANOVA with Tukey’s test. Table S1: Primers used in this work.
