# Supplementary material for: MaCsbD Mediates Thermotolerance and UV-B Resistance in Metarhizium acridum by Regulating DNA Repair, Antioxidant Defense, and Protective Metabolites
Source: J Fungi (Basel). 2025 Nov 27;11(12):838. doi: 10.3390/jof11120838 (PMC12733939; doi:10.3390/jof11120838)
Supplement: Supplementary file 1 [file jof-11-00838-s001.zip › Figure S1.pdf]

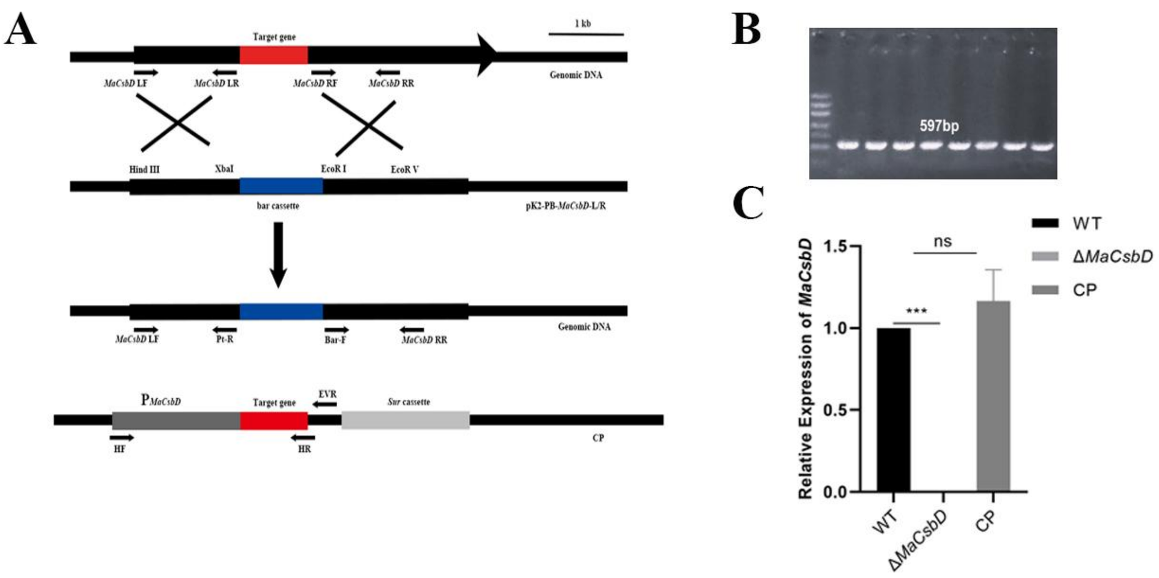

Figure S1: The disruption and complementation of *MaCsbD*. (**A**) The knockout and complement schematic diagram of *MaCsbD*. (**B**) PCR was used to verify the complementary strains of *MaCsbD*. (**C**) Transcription-level analysis of *MaCsbD* in WT, Δ*MaCsbD*, and CP strains. Asterisks indicate significant differences (*** p < 0.001; ns, not significant) as determined by one-way ANOVA with Tukey’s test..
