# Supplementary material for: MaCsbD Mediates Thermotolerance and UV-B Resistance in Metarhizium acridum by Regulating DNA Repair, Antioxidant Defense, and Protective Metabolites
Source: J Fungi (Basel). 2025 Nov 27;11(12):838. doi: 10.3390/jof11120838 (PMC12733939; doi:10.3390/jof11120838)
Supplement: Supplementary file 1 [file jof-11-00838-s001.zip › Table S1. Primers used in this work.pdf]

Primers	Sequences (5′- 3′)	Remarks
MaCsbD-LE	AAGAAAGCCGAAGATTCAATTGGG	Used to clone the 5′ end of MaCsbD
MaCsbD-LR	AGAGTGAGTTGGCAAGAGTTGA	
MaCsbD-RF	AGTCTTGCTAAATGGCTTTCCCT	Used to clone the 3′ end of MaCsbD
MaCsbD-RR	AACCGCCAAACGAACCATCA	
Pt-R	CAGCCAAGCCCAAAAAGTG	Used for screening the MaCsbD - disruption transformants
Bar-F	GCTCTACACCCACCTGCT	
MaCsbD-VF	ACTGGAGAGCGACTCTCG	
MaCsbD-VR	GGACAGCGTCAAAGAAGA	
MaCsbD-CPF	AAGACTACGAATACTTCAATGACC	Used to form pK2-MaCsbD -EGFP-SUR vector
MaCsbD-CPR	TTCTTTCTTTTGCTCAGCCTC	
CP-VF	GTTGGCAGTGAGGTAAGTTGAC	Used for screening the MaCsbD - complement transformants
EGFP-VR	AAGAAGTCGTGCTGCTTCATGTG	
Gapdh-qF	AGATGGAGGAGTTGGTGTTG	Used to analyze the expression of MaCsbD by qRT-PCR
Gapdh-qR	GACTGCCCGCATTGAGAAG	
MaCsbD-qF	AACTGGAATCAGACCGTTGG	
MaCsbD-qR	TGATGTTGGAAATGGCACCT	
qPksP-F	GGCCTGCAGGAAGTCATTCT	Used to verify the transcriptional levels of genes related to melanin biosynthesis-
qPksP-R	ATGAGGAAGCCCGTCAGTTG	
qSCD-F	TGGTTGCTGCTGGGATACTG	
qSCD-R	AGAGTGTTGGTGCGAGGATG	
qTHR-F	GGCATGCCCATGGAAGAGAT	
qTHR-R	TCACCCACTCGCTATCAGGA	
qAbr1-F	TCAACTACAACTCCACCGGC	
qAbr1-R	GTAGTTGGCACCATCACCGA	
qLAC-F	CGGAGCCGGTAAGTGGAAAT	
qLAC-R	GGTTTGTGACCTGGTAGCGA	
qTyr-F	TCCTCTGAACGGGCTAGTCA	
qTyr-R	ACCAGTAAGCTTGGACACGG	
qHppD-F	GATGTGGCCTTTGAGGTGGA	
qHppD-R	GACTTCTTGCCCTCCCGAAA	
qGPX-F	TCGCTGACTACAAGGGCAAG	Used to verify the transcriptional levels of genes related to ROS scavenging
qGPX-R	CTGCATGATGGGGAAGCTGA	
qSOD-1-F	CATGCCTATTACCTACAGTA	
qSOD-1-R	CTTCCAGTTGATGACCTT	
qSOD-2-F	AGACAAGGACACCAAGAC	
qSOD-2-R	GGAGGCGTAGTTGAAGAT	
qSOD-4-F	GGTCAAAGCTGTTGCCGTTC	
qSOD-4-R	CGCCAAAGGTGTGAATGTGG	
qMnSOD-F	CTCCTCTTCTGAACTTCC	
qMnSOD-R	CCGAAATCCTCATTGATG	

qCAT-1-F	GATACGGTAACAGCAATGG	
qCAT-1-R	TCGGTCAAGTCAGATACG	
qCAT-2-F	TTCTCGCCATCCAATCTC	
qCAT-2-R	TCGTCGTTATTCCGTCTC	
qCAT-3-F	CCTCTACTCTTCATACCTTGT	
qCAT-3-R	GTCGCCATTCTCATTCAC	
qCAT-4-F	AAGTTCCCGTTGTTCATC	
qCAT-4-R	TTCCTGGTGAGTTGAGAG	
qPOD-F	TGTCGTGTCCAACCTGTACG	
qPOD-R	CGCTGTTGGCGATAATGACG	
qRAD2-F	AGGAAGTGGTAGTGGCAACG	
qRAD2-R	GAGCTGCTGAAACTGAGGGT	
qRAD3-F	ATCACTCCTGACGGCCTAGT	
qRAD3-R	CTTCTGAGACTTTGCCCCGT	
qRAD4-F	AAATGCGAAGGAAGCAGGGA	
qRAD4-R	TCGAACGAGTTGCTGCAGAT	
qRAD10-F	CAGCCCGTACCCCAAAGAAT	
qRAD10-R	GCAGGTTGTGAGTCCCATGA	
qRAD14-F	ACATGATGCTGTTCCTGCGA	
qRAD14-R	TCTGTTCGAGTCTTGCGCTT	
qRAD16-F	AAACACGACTTTTGCCGAGC	
qRAD16-R	ATGCGTTGTCTGAACGGAGT	
qRAD23-F	AGGAAGTGGTAGTGGCAACG	Used to verify the transcriptional expression of genes in NER pathway
qRAD23-R	GAGCTGCTGAAACTGAGGGT	
qRAD25-F	CAAGAGGTCACGCACTCCTT	
qRAD25-R	CCACTCTTGGCTCGACCATT	
qHSP40-1-F	GGCGAAGCGGATCAATTTCC	
qHSP40-1-R	CATACCTTCGCCAGCGATCT	
qHSP40-2-F	CACGACCGAGTTTCAGTGGA	
qHSP40-2-R	TGAGAGTGACAGTCGTTGGC	
qHSP70-1-F	TGTCGATTTGGGCACCACTT	
qHSP70-1-R	GTAGACAATGGCGGTTGGGA	
qHSP70-2-F	GCTTGATGTTGCCCCTCTCT	
qHSP70-2-R	TGACGCTCACCCTCAAAGAC	
qHSP70-3-F	AGTCCTACATTGGCCGTGTG	
qHSP70-3-R	CTTAAGGGCAAGCTCCTGCT	
qHSP90-F	AAGAAGGTTGAGGCTGACGG	
qHSP90-R	GAGCTTGTGAATGCGCTCAG	
